# Note on Posterior Nasal Tampon

**Published:** 1895-12

**Authors:** John T. Pitkin

**Affiliations:** Buffalo, N. Y.


### NOTE ON POSTERIOR NASAL TAMPON.

By JOHN T. PITKIN, M. D., Buffalo, N. Y.

FOR the arrest of severe epistaxis by a posterior nasal tampon, on account of its simplicity of performance and absolute results obtainable therefrom, I would solicit the reader's attention to my *modus operandi*.

With the patient seated, preferably in a stiff-back chair, insert one end of a small-sized elastic rubber tube with a strong silk cord passing through its lumen (tube should be from two and a half to three feet long), into the bleeding nostril. When from three to five inches have been thus introduced, instruct the patient to make repeated forced expiratory efforts through the mouth, *i. e.*, cough, while the operator continues the slow introduction of more tubing *via* nares. In a few moments the distal end of the tube, with its contained cord, will be extruded from the buccal cavity. Should vomiting intervene, although otherwise by no means desirable, nevertheless it would accomplish the same desideratum. Now, if a medicated cotton tampon is securely fastened to the mouth end of the cord, and gentle traction made upon its nasal extremity, the tampon will be drawn to the position desired in the posterior nares. The rubber tube can then be slipped off of the cord, which, with the pledget of cotton, will be left *in situ*.

